# Long-Term Effects on Loneliness of a Computer-Tailored Intervention for Older Adults With Chronic Diseases: A Randomized Controlled Trial

**DOI:** 10.1177/08982643211015027

**Published:** 2021-05-07

**Authors:** Janet M. Boekhout, Esmee Volders, Catherine A. W. Bolman, Renate H. M. de Groot, Lilian Lechner

**Affiliations:** 1 Faculty of Psychology, 10198 Open University of The Netherlands, Heerlen, The Netherlands; 2 Faculty of Educational Sciences, 10198 Open University of The Netherlands, Heerlen, The Netherlands; 3 Nutrition and Translational Research in Metabolism (School NUTRIM), 5211 Maastricht University, Limburg, The Netherlands

**Keywords:** loneliness, older adults, chronic diseases, mobility impairments

## Abstract

**Objectives:** This study explores the effects of the Active Plus intervention aiming to decrease loneliness among older adults (>65 years) with chronic diseases. **Methods:** A randomized controlled trial (RCT) was performed *(N* = 585; age: *M* = 74.5 years, *SD* = 6.4), assessing loneliness at baseline, 6 months and 12 months. Outcome measures in the multilevel linear regression analyses were total, social and emotional loneliness. **Results:** At 12 months, significant decreases in total (*B* = −.37, *p* = .01) and social loneliness (*B* = −.24, *p* = .02) were found. Age was a significant moderator for total and social loneliness; however, the intervention was effective only for participants aged 80 years and older. **Discussion:** The Active Plus intervention showed a significant decrease in total and social loneliness and was especially beneficial for the vulnerable age group of 80 years and older. A more comprehensive tool for measuring social activity and mobility impairments, and using a longer time frame to detect loneliness changes, may form interesting future research.

## Introduction

Most countries in the world are faced with demographic distributions that shift towards older ages. In 2019, 1 in 10 people was aged 65 years or over, which is projected to increase to 1 in 6 people by 2050 (United Nations, 2019). In high-income countries, the portion of older adults (>65 years) in this distribution is even higher (He et al., 2016). Older age is not only accompanied with a decline in health but also with life transitions, such as retirement from working life and the loss of a spouse (Holt-Lunstad, 2018; National Academies of Sciences, Engineering, and Medicine, 2020; World Health Organisation, 2018b). These changes clear the path for the onset and continuance of loneliness, especially for older adults with chronic diseases (van Hees et al., 2020; Wrzus et al., 2013). As loneliness has been found to be a risk factor for mortality almost equally detrimental as smoking, obesity or physical inactivity (Holt-Lunstad et al., 2015), interventions to reduce loneliness among the most vulnerable groups of older adults are called for.

An often used definition for loneliness is ‘the unpleasant experience or feelings associated with a lack of close relationships’ (de Jong-Gierveld, 1998). This definition demonstrates that loneliness is a qualitative appraisal rather than an objective state: the size of one’s social network is subordinated to how the relationships within that network are valued (Cohen-Mansfield & Perach, 2015; de Jong-Gierveld, 1998; McHugh Power et al., 2018). Loneliness is often considered a bi-dimensional construct consisting of social and emotional loneliness (Dahlberg & McKee, 2014; Dutch Central Bureau of Statistics, 2018; McHugh Power et al., 2018). Social loneliness refers to the perceived absence of a social network, such as a circle of friends or acquaintances that fulfil a need of belonging, while emotional loneliness refers to the perceived absence of an intimate partner or close friend who provides a feeling of close attachment (Dahlberg & McKee, 2014; de Jong-Gierveld, 1998).

Both the prevalence and severity of loneliness increase with age. In the age group of 65–74 years, 44% reports being lonely, rising to 53% in the age group of 75–84 years and 63% for those of 85 years and older (Dutch Central Bureau of Statistics, 2016); these figures are in line with other high-income countries (European Union, 2015; National Academies of Sciences, Engineering and Medicine, 2020). Loneliness is more prevalent, and more severe, in older adults with a chronic disease, than in those without (Meek et al., 2018; Richard et al., 2017).

Loneliness has been found to be closely related to many aspects of health (Holt-Lunstad et al., 2015; Rico-Uribe et al., 2016; Tilvis et al., 2011; Valtorta et al., 2016). Physical health is affected as older adults not only experience an overall decline of physical abilities with age, but the majority of older adults develop one or more chronic diseases, such as type 2 diabetes, arthritis, cardiovascular diseases or chronic obstructive pulmonary disease (COPD) (Courtin & Knapp, 2017; Leigh-Hunt et al., 2017; Luo et al., 2012; Ong et al., 2016; The Lancet, 2020). Moreover, the prevalence of chronic diseases increases with age: in the age category of 65–74 years, 82% of the Dutch population has a chronic disease, which increases to 90% from the age of 75 years and over (Dutch Department of Health Wellbeing and Sports, 2016a); other high-income countries show similar numbers (European Union, 2015; World Health Organisation, 2018a).

Although literature shows no uniform definition of what a chronic disease is, most definitions include the presence of some form of mobility impairment (Goodman et al., 2013; McKenna et al., 2010). These mobility impairments potentially threaten social and mental health as research has consistently shown that mobility impairments are associated with diminished social participation (Everard et al., 2000; Mendes de Leon et al., 2003; Puts et al., 2007), with higher feelings of loneliness (Griffith et al., 2017; Nicolaisen & Thorsen, 2012; Smith & Victor, 2018; van Hees et al., 2020) and with depression or anxiety (Adams et al., 2004; Cacioppo & Cacioppo, 2014; Global Council on Brain Health, 2017; Heinrich & Gullone, 2006). A negative spiral may occur as lonely individuals tend to withdraw increasingly from social life (Asante & Castillo, 2018; Cohen-Mansfield et al., 2016; Courtin & Knapp, 2017).

In addition to these health issues, several societal changes increase the risk of loneliness for older adults. Due to the budget limitations in social care, older adults are stimulated to live independently at home for as long as possible instead of moving into a retirement home, resulting in more and more older adults living alone (Dutch Department of Health Wellbeing and Sports, 2018; Valtorta & Hanratty, 2012). Conversely, due to the decline in physical and cognitive functioning that is accompanied with older age, a decrease in daily activities and societal participation may be seen which for those living alone is especially challenging (de Hond et al., 2019; Tak et al., 2013).

The increasing prevalence of loneliness and severity of related health risks have been the basis for the increased societal and academic interest in preventing and alleviating loneliness among older adults, making it a major target in governmental public health policies worldwide (World Health Organisation, 2018b). Although this is substantiated by the number and variety of interventions targeting loneliness in older adults (e.g. improving social skills, enhancing social support, increasing opportunities for social contact and addressing maladaptive social cognition) (Courtin & Knapp, 2017; Fakoya et al., 2020; Jarvis et al., 2020), there still seems to be a dearth in research focussing on alleviating loneliness among older adults with mobility impairments caused by chronic diseases, a target population that is especially vulnerable for loneliness (Petitte et al., 2015; Poscia et al., 2018).

In this study, we examine the effects of the Active Plus intervention on loneliness among that specific target population. This computer-tailored intervention was originally developed for the general public of 50 years and over (Peels et al., 2012) and has later been adapted for the specific target population of older adults with chronic diseases (Boekhout et al., 2017; Volders et al., 2019). The intervention aims primarily to increase physical activity (PA) and cognitive functioning and secondarily to decrease loneliness by offering a computer-tailored advice. The development of the intervention (Boekhout et al., 2017; Volders et al., 2019) and the limited effects on PA (Volders et al., 2020) have been described previously. The computer-tailored advice emphasizes the importance of social connectedness and suggests ways to increase social activity while being physically active, in order to decrease loneliness. The negative association between social activity and loneliness has often been described in the literature (Bruggencate et al., 2018; Cohen-Mansfield & Perach, 2015; Gardiner et al., 2018), and as such, stimulating social activity in order to decrease loneliness is an often used approach in interventions (Dickens et al., 2011; Robins et al., 2016a, 2016b).

A previously performed quasi experimental study by Boekhout et al. (2019) into the effects of Active Plus on loneliness showed a decreased total loneliness among the participants of the intervention (i.e. single older adults with mobility impairments). Considering the often cited meta-analysis of Masi et al. (2011), showing that social activity is less suited to alleviate emotional loneliness, we mainly expect to find effects on total and social loneliness. In addition, exploratory analyses for a potential moderating role of gender, marital status, age, degree of impairment and educational attainment will be performed as a recent study by van Hees et al. (2020) demonstrated that these demographics were associated with loneliness.

## Methods

### Study Design

This study is part of clustered randomized controlled intervention trial (RCT) into the efficacy of the Active Plus intervention. Active Plus primarily aims to stimulate cognitive functioning and PA and secondarily to decrease loneliness. The intervention is developed for the target population of older adults, independently living in the community, with chronic diseases. The rationale and description of the study protocol has previously been described extensively (Volders et al., 2019).

For this study, a clustered two-group RCT was performed, in which participants were allocated to either the Active Plus intervention group or to a waiting list control group, with assessments at baseline, 6 months and 12 months. The study was conducted following the Declaration of Helsinki (World Medical Association, 2013). All participants provided written informed consent.

### Procedure and Participants

**Figure 1.**
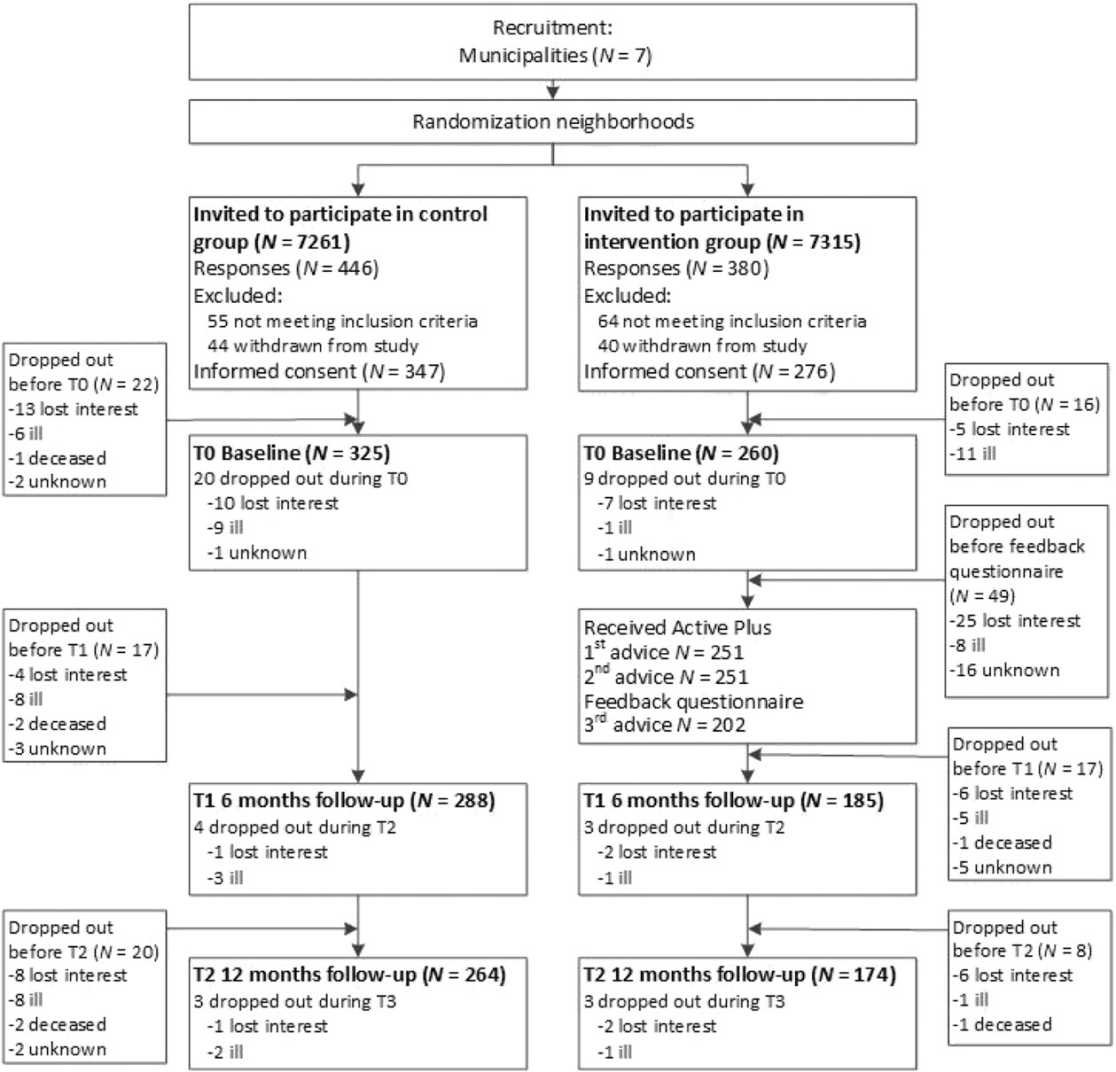
Flow chart of the study.

Participants were recruited through seven municipalities in the Netherlands that agreed to participate in this RCT. As these municipalities in themselves are not comparable regarding socio-economic status, randomization was done on a neighbourhood level within each municipality. The municipalities each selected two neighbourhoods with comparable socio-economic statuses (Association of Dutch Municipalities, 2020) that were randomly assigned to either an intervention group neighbourhood or a control group neighbourhood (ratio 1:1). Randomization was performed by the researchers by means of online randomizer software (Haahr, 2020). Per neighbourhood, the municipalities sent direct postal mailings to 250–2000 addresses of independently living older adults (aged 65 years or older). The mailing consisted of a personalized information letter and a prepaid response card including informed consent that could be returned to the researchers. Enrolment lasted from February to July 2018. Inclusion criteria were 65 years or older, fluent in Dutch, having at least one mobility affecting chronic disease (e.g., COPD), arthritis, osteoporosis, chronic heart disease) or other mobility affecting physical condition (e.g., visual or hearing impairments). Exclusion criteria were severe self-reported cognitive impairments, using a wheelchair or not being able to walk at least 100 m (with or without the help of a walker or walking stick). Both the intervention group and waiting list control group received a paper questionnaire with a prepaid return envelope as well as access codes for the Active Plus website where the questionnaire could be completed online, giving the participant a choice between these two delivery modes (i.e. paper or online). The 4-month lasting intervention then commenced for the intervention group. The second and third questionnaire followed after 6 and 12 months in the same procedure as the baseline measurement. The participants in the waiting list control group were given access to the Active Plus intervention directly after the 12 months assessment. Next to filling in the questionnaires, participants also wore accelerometers to assess PA and took cognitive functioning tests on computer/tablets in the presence of researchers. As these assessments are not part of the present study, these assessments are not described here in further detail: more information can be found in Volders et al. (2019). Figure 1 presents the flow chart of the study.

### Intervention

Active Plus is a systematically developed computer-tailored intervention, which was adapted for independently living older adults (65+ years) with chronic diseases. The intervention provides participants with three individualized PA advices (delivered by paper and online) over a time frame of 4 months. These advices are based on 2 questionnaires (also delivered by paper and online) that were filled in on baseline and after 3 months. The first advice, based on the baseline questionnaire, addresses mainly pre-motivational psychosocial constructs such as awareness raising of the benefits of being physically active with other people and informing people about exercise clubs for their age group or chronic disease that are available in the local municipality. The second advice, also based on the baseline questionnaire, focuses on motivational psychosocial constructs such as increasing the perceived self-efficacy and attitude towards making new social contacts for enacting in PA: this is done, for example, by explaining that for everyone attending an exercise club for the first time with unfamiliar people, it may feel awkward at first but that generally everyone is accepted in a social and friendly way. In the third advice, based on the second questionnaire, post-motivational psychosocial constructs are targeted, such as action planning: this is, for example, done by showing a prefilled week calendar that suggests activities such as ‘walking the dog(s) together with my neighbour’. Depending on the stage of change the participant is in, the focus of the individual advice may shift.

The advice is tailored to the individual’s needs by computer tailoring. In this tailoring, the participants’ demographics, stages of change, psychosocial characteristics, and their degree of mobility impairment are considered. For example, a female participant in the contemplation stage of becoming more active may be shown a role model video of a woman in the same age category who tells about the benefits that being more active has brought her; a male participant who is in the preparation stage may be shown a comparable role model story of a man in his age category but then with an emphasis on finding social support for becoming more active. Another example is that a participant with a mild degree of arthritis in the lower limbs may receive the advice to join a swimming club, whereas a participant with a high degree of arthritis is the upper limbs may receive an advice to join a walking club. The advice is presented in a mainly text-based format (for both the paper and online delivery method) which is complemented with graphic materials like charts, pictures (written delivery mode) and videos (online delivery mode). As the intervention is implemented by municipalities or even in specific neighbourhoods, extensive information on physical or social activities that are available locally has been added to the advice.

## Measures

### Loneliness

Loneliness is assessed by the 6-item De Jong Gierveld Loneliness Scale. This self-report questionnaire is widely used in Europe, and its psychometric qualities are considered acceptable with a scale reliability ranging between .80 and .90 Cronbach’s alpha and a scale homogeneity ranging between .30 and .50 (Gierveld & Tilburg, 2006). The scale has three items evaluating social loneliness (e.g. ‘There are plenty of people I can rely on when I have problems’) and three items evaluating emotional loneliness (e.g. ‘I experience a general feeling of emptiness’). These subscales can be combined to a total loneliness score. The original 5-point scale of this questionnaire (i.e. yes!, yes, more or less, no, no!) was adapted to a 10-point scale (1 = absolutely not and 10 = absolutely sure) to better align the scale with the target population’s preference for distinct words instead of exclamation marks and for comparable point scales (Krosnick and Presser, 2010; Remillard et al., 2014). Similar to the data handling as described in the manual (de Jong Gierveld & Van Tilburg, 1999), after recoding the items in the correct direction, answers from 1 to 5 represent no loneliness, and answers from 6 to 10 represent loneliness. When one item or more is not answered, no score for social or emotional loneliness can be calculated: for the total score of loneliness, a maximum of one item may be missing. The mean is then taken for answers that indicate loneliness, resulting in a potential score of loneliness between 0 and 3 for the social and emotional subscales and between 0 and 6 for the total loneliness score, with higher scores indicating more loneliness.

### Demographics

Several demographics were corrected for in the analyses as these are known to affect loneliness (Gouveia et al., 2017; Ku et al., 2016; Shvedko et al., 2017; van Hees et al., 2020), including age (in years), gender (0 = male/1 = female), marital status (1 = living alone/2 = living with spouse), educational attainment, body mass index (BMI) (length in metres divided by squared weight in kilograms) and degree of mobility impairments. Educational attainment was categorized into low (i.e. primary, basic vocational or lower general level = 1), moderate (i.e. medium vocational, higher general secondary or preparatory academic level = 2) or high (higher vocational or university level = 3). The degree of the mobility impairment was assessed with 15 items, 14 for most prevalent chronic diseases (i.e. COPD and arthritis) or physical conditions (i.e. visual or hearing impairments) and 1 for any other chronic diseases not mentioned. For each item, participants could indicate the degree of mobility impairment on a 5-point scale (ranging from 0 = not impaired to 4 = severely impaired). The degree of impairment was categorized into three categories being 1 = little impaired (with a maximum score of 1 on at least one item), 2 = medium impaired (with a maximum score of 2 on at least one item) and 3 = severely impaired (with at least a score of 3 or 4 on at least one item).

### Analyses

Baseline differences between the intervention and control groups were analysed by chi-square tests (categorical variables), Mann–Whitney U-tests (skewed continuous variables) and ANOVA tests (non-skewed continuous variables). Binary logistic regression was conducted to assess selective dropout at 6 and 12 months.

As measurement points are nested within the participants, and with participants originating from different municipalities, a potential interdependence was present. Therefore, multilevel linear regression analyses were performed with measurement points as level 1, participants as level 2 and municipality as level 3. The analyses demonstrated that the intraclass coefficients (ICC) for the dependent variables of social loneliness, emotional loneliness and total loneliness were all smaller than .01. As a result, two-level analyses were performed. Participants were included as random effect in the model; measurement points, group and the interaction between measurement points and group were included in the models as fixed effects to assess the intervention effects over time. Intervention effects between the intervention group and control group were compared between baseline and 6 months follow-up and between baseline and 12 months follow-up. For all analyses, age, gender, educational level, marital status, BMI and degree of impairment were added as covariates. Continuous variables were standardized. Confidence intervals (CIs) were calculated for all outcomes. Analyses were conducted on an intention-to-treat basis without any ad hoc imputation (Twisk et al., 2013).

Exploratory differences regarding intervention efficacy were assessed for degree of impairment, age, gender, educational level, marital status and BMI. Three-way interaction terms (time x group x covariate) of significant covariates were added to the model. When a three-way interaction term was significant, subgroup effects were examined by repeating the analyses. In these multilevel analyses, the two-level data structure was applied again. Subgroups were defined by the categories of the covariates for categorical variables. For the continuous variables of age and BMI, the groups were split at, respectively, 80 years or older or 79 years and younger and at obese or non-obese (limit at 30 kg/m^2^).

All analyses were performed with R (R Core Team, 2019). In all analyses, a reproducibility level of 95% was applied (α =.05). Since interaction terms have less power, the significance levels were set to *p* < .10 for the interaction terms. Sample size was found to be sufficient, based on sample size calculations that were performed a priori on the primary outcome measures of the intervention, and are described elaborately elsewhere (Volders et al., 2019).

## Results

### Study Population

A total of 623 participants provided informed consent and were included in the study (see Figure 1). Before baseline, 38 withdrew, resulting in 585 participants at baseline (age: *M* = 74.5 years, *SD* = 6.4), with an almost equal gender distribution (48.4% men). Living with a spouse was the most prevalent marital status (80.7%). Regarding educational attainment, 51.2% was low educated. Most participants (51.2%) were medium impaired. No significant baseline differences were found between the intervention and control groups (see Table 1). A subgroup analysis for loneliness, where the groups were split in younger than 80 years and 80 years and over, however, showed that the intervention group was significantly more lonely than the control group (see Table 1).

**Table 1.** Baseline Participant Characteristics of The Control Group and The Intervention Group.

	Control group (*N* = 325)	Intervention group (*N* = 260)	*p* value
Demographic characteristics			
Age in years, mean (*SD*)	74.46 (6.22)	74.20 (6.60)	.62
Gender, *N* (%)			.59
Male	164 (50.5%)	138 (53.1%)	
Female	161 (49.5%)	122 (46.9%)	
Marital status, *N* (%)			.09
Living single	50 (16.6%)	56 (22.6%)	
Living together	252 (83.4%)	192 (77.4%)	
Education, *N* (%)			.54
Low	151 (50.3%)	127 (52.3%)	
Middle	60 (20.0%)	54 (22.2%)	
High	89 (29.7%)	62 (25.5%)	
Health-related characteristics			.35
BMI, median (IQR)*	26.9 (24.1–29.4)	26.9 (24.4–29.8)	
Degree of impairment, *N* (%)			.39
Little impaired	34 (11.1%)	29 (11.6%)	
Medium impaired	134 (43.8%)	123 (49.0%)	
Severely impaired	138 (45.1%)	99 (39.4%)	
Loneliness, *N*	305	251	
Total loneliness, mean (*SD*); %	1.84 (1.80); 51.5**	2.02 (1.94); 51.3	.27***
Social loneliness, mean (*SD*); %	.83 (1.12); 42.2	.95 (1.23); 43.8	.23
Emotional loneliness, mean (*SD*); %	1.00 (1.19); 48.5	1.06 (1.20); 51.8	.55

*Notes. SD* = standard deviation; IQR = interquartile range; BMI = body mass index. *non-normally distributed variable tested with Mann–Whitney *U* test. **percentage of total intervention or control group that reports being lonely; ***p value for differences in mean.

Dropout at 6 months and 12 months was, respectively, 19.1% (112/585) and 25.1% (147/585). Participating in the intervention group (6 months: *OR* = 5.85, 95% *CI* = 3.38; 10.56, *p* ≤ .001; 12 months: *OR* = 2.72, 95% *CI* = 1.76; 4.25, *p* ≤ .001) and older age (6 months: *OR* = 1.09, 95% *CI* = 1.05; 1.14, *p* ≤ .001; 12 months: *OR* = 1.08, 95% *CI* = 1.04; 1.12, *p* ≤ .001) were predictors of dropout at both 6 and 12 months after baseline. In addition, a low education was a predictor of dropout at 12 months (*OR* = 2.03, 95% *CI* = 1.16; 3.66, *p* = .015). In the control group, the most frequent reason for dropout (30 out of 64 dropouts) was being too ill to continue, while intervention group participants mostly (48 out of 89 dropouts) dropped out due to a loss of interest. For the outcome measure of loneliness, 29 participants of the total of 585 had a missing baseline measurement of loneliness: in the analyses at 6 and 12 months, an additional 21 participants were missing and could not be included in the analyses.

### Intervention Effects

Table 2 shows the intervention effects on loneliness. 12 months after baseline, participants in the Active Plus group scored significantly lower on total loneliness (*B* = −.37, *SE* = .15, *p* = .01) and social loneliness (*B* = −.24, *SE* = .10, *p* = .02) after adjusting for potential confounders, indicating less loneliness in the intervention group. No significant differences between the intervention and control groups were found in emotional loneliness.

**Table 2.** Intervention Effects (Group x Time Interaction) on Loneliness Outcomes for 6 and 12 Months Follow-Up*.

		Effect after 6 months				Effect after 12 months			
	*N*	*B*	*SE*	95%*CI*	*P*	*B*	*SE*	95%*CI*	*p*
Loneliness									
Total loneliness	535	−.17	.15	−.47; .12	.24	−.37	.15	−.67; −.08	.01
Social loneliness	535	−.13	.10	−.32; .06	.19	−.24	.10	−.43; −.04	.02
Emotional loneliness	535	−.05	.11	−.26; .16	.67	−.14	.11	−.35; .08	.21

*Notes. SE =* standard error; CI = confidence interval. *Effects are reported as intervention group versus control group as control group served as reference group adjusted for covariates.

### Moderation Effects

Although the intervention is individually tailored, it is possible that not all subsets of participants react similar to the intervention. To explore this, analyses for subgroups were performed. These exploratory analyses (see Table 3) showed that only for age a significant moderation effect (intervention group vs. control group) was present. Intervention group participants of 80 years or older had significantly lower total loneliness scores at both 6 months after baseline (*B* = −.82, *p* = .03) and at 12 months after baseline (*B* = −0.76, *p* = .05) compared to control group participants of 80 years or older. However, the intervention effect was not present in participants younger than 80 years (6m: *B* = −.01, *p* = .95; 12m: *B* = −.26, *p* = .11). This moderation effect of age was also present in social loneliness, where the intervention was only effective for participants of 80 years or older (6m: *B* = −.60, *p* = .01; 12m: *B* = −.57, *p* = .02), as opposed to participants younger than 80 years (*B* = −.01, *p* = .94; 12m: *B* = −.15, *p* = .19). For emotional loneliness, no moderation effect was found.

**Table 3.** Moderation of Intervention Effects (Group x Time Interaction) on Loneliness Outcomes for 6 and 12 Months Follow-Up in Subgroups*.

		Effect after 12 months					Effect after 12 months			
	Subgroup	*N*	*B*	*SE*	*CI*	*p*	*B*	*SE*	*CI*	*p*
Total loneliness	≥80 years	114	−.82	.37	−1.55;−.09	.03	−.76	.39	−1.53; .00	.05
	<80 years	421	−.01	.16	−.32; .31	.95	−.26	.16	−.58; .06	.11
Social loneliness	≥80 years	114	−.60	.22	−1.04;−.17	.01	−.57	.24	−1.03;−.11	.02
	<80 years	421	−.01	.11	−.22; .21	.94	−.15	.11	−.36; .07	.19

*Notes. SE* = standard error; CI = confidence interval. *Effects are reported as intervention group versus control group as control group served as reference group in the different subgroups adjusted for covariates.

## Discussion

This study investigated the effects of the Active Plus intervention on loneliness among the target population of independently living older adults with chronic diseases. At 12 months, a significant intervention effect was found for total loneliness and for social loneliness but not for emotional loneliness. Age was a significant moderator for total and social loneliness at 6 and 12 months: only in the age group of 80 years and over, the Active Plus intervention was effective.

The significant effect that we found on total loneliness is in line with several reviews demonstrating that stimulating social activity while being physically active, which the Active Plus intervention does, can contribute to alleviating loneliness (Dickens et al., 2011; Robins et al., 2016a, 2016b). Several characteristics of the Active Plus intervention, such as being developed within the context of a theoretical basis (Dickens et al., 2011), integration in the community setting and stimulation of participation in local activities (Gardiner et al., 2018), are known to increase the effects of these types of interventions and can therefore contribute to our findings. However, not all previous research distinguishes between social and emotional loneliness, which is important to take into consideration. For our intervention, as expected, only social loneliness showed a significant effect. Although many loneliness interventions employ social activity as a means to decrease overall loneliness (Asante & Castillo, 2018; Courtin & Knapp, 2017), some studies have suggested that stimulating social activity has the potential to decrease social loneliness but is not an appropriate method to decrease emotional loneliness (Machielse, 2015; Masi et al., 2011; O’Rourke et al., 2018). By increasing social activity, individuals may acquire more social contacts and thus decrease their social loneliness, but an increase in the quantity of social contacts does not necessarily mean that these contacts provide a deep emotional bond, which is a prerequisite for decreasing emotional loneliness. That we did find an effect on social but not on emotional loneliness is thus as expected, considering the design of the intervention.

When interpreting our results, it is important to take into consideration the characteristics of our intervention group of whom 43% was medium impaired and 45% was severely impaired. Severe mobility impairments are known to impede the potential to be socially active, for example, when they restrict access to private or public transport and thus limit the possibilities to join social activities or to visit others (Dahlberg et al., 2015; Robins et al., 2016a, 2016b). This is also corroborated by a 5-year longitudinal study (Newall et al., 2014) who showed that decreases in loneliness were only seen in those who perceived a change of control over their life situation: as severe mobility impairments are known to negatively affect feelings of control (Barlow et al., 2015; Hawkley & Cacioppo, 2010), this could implicate that loneliness is very difficult to decrease for those with higher degrees of mobility impairments. This may indicate that the fact that our intervention did show effects among this group of participants is particularly auspicious. A potential implication for practice may be that future interventions need to stronger address possibilities of being socially active when mobility impairments are present. For future interventions, it may also be useful to carefully consider which measurement instrument is used to determine the degree in which social activity is impeded by mobility impairments. Although validated instruments exist, they may not be suitable for the group of independently living older adults with mobility impairments: these instruments are often designed for the more fragile who require substantial help from others in daily life (Ustün et al., 2010; Ware et al., 1995). A more precise measurement instrument for measuring the degree in which social activity is impeded by the degree of mobility impairments or by the type and features of a specific chronic disease may enhance the computer tailoring of the advice and therefore also the effects of the intervention.

Our findings may also be explained by age differences in normative expectations regarding loneliness. Due to normative expectations, individuals in their twenties, for example, may feel lonely when having only two close friends as in that age group the perceived norm is to have a large group of friends, whereas individuals in their seventies or eighties may feel blessed for still having one or two close friends (Luhmann & Hawkley, 2016). These normative expectations may thus result in a lower incentive among older adults to actively intensify or expand present close contacts. Also, for older people, the quality instead of the quantity of social contacts is more negatively related to loneliness (Green et al., 2001; Victor & Yang, 2012): as it takes time to acquire contacts of a certain quality, emotional loneliness is harder to change than social loneliness. This may also explain why for the entire intervention group, no intervention effects for either social, emotional or total loneliness were found at 6 months. A recent meta-analysis has shown that loneliness has trait-like features making it a relatively stable sensation for older adults that may stay present fairly long and irrespective of changes in their current circumstances (Mund et al., 2020). It may therefore be that changes in loneliness are better assessed after a longer time frame than done in our study.

Only age and marital status were significantly related to loneliness: age was positively associated with total and emotional loneliness (i.e. the older, the more lonely), and living alone was associated with more emotional loneliness. A recent study by van Hees et al. (2020) demonstrated that being older, living alone, the male gender, being more disabled and being lower educated were associated with higher levels of loneliness. As in our sample, most participants have medium to severe mobility impairment and the large majority is lower educated, our findings are not very divergent from that study, with only gender showing no significant relation to loneliness. Age proved to be the sole significant moderator: only in the age group of 80 years and over, the Active Plus intervention was effective in decreasing total and social loneliness, both at 6 and at 12 months. An explanation may be found in the prevalence of loneliness which of all age groups is the highest in the oldest age groups. In our sample, loneliness was significantly higher in the age group of 80 years and older than in the age group younger than 80 years (total loneliness 62.7% vs. 48.4%; social loneliness 45.0% vs. 42.3%; emotional loneliness 63.3% vs. 47.3%). These figures are in line with national prevalence data showing that 44% of older adults between 65 and 74 years report being lonely, increasing to 63% in those over 85 years (Dutch Department of Health Wellbeing and Sports, 2016b). Qualter et al. (2015) suggest that interventions that focus on alleviating loneliness by stimulating social activity may be especially beneficial to the most vulnerable groups where loneliness is already higher, such as the oldest old. This age group is generally less able to connect with others as they have fewer opportunities to engage socially. As the Active Plus intervention offers these opportunities by suggesting social activities that are easy to integrate in daily life, it may therefore be especially suited to decrease social loneliness for the vulnerable group of the oldest old. For the future, this suitability may even be enhanced by incorporating into the computer-tailored advice what social activities the participants find the most meaningful: it has been argued that interventions to decrease loneliness are more effective when people are linked with others and with activities they find meaningful (Pels & Kleinert, 2016; Steffens et al., 2016). A study on the use, appreciation and working mechanisms of the intervention may also shine a light on what variables need extra attention in the computer tailoring of advice and would thus form an interesting line of future research.

### Methodological Issues

Although our study provides relevant insights derived from a study with a methodological vigorous design, some methodological issues should be noted. First of all, the initial response rate of 6% is relatively low, although meaningful comparisons are difficult to make as most studies report only on dropout and not on response rates (Zubala et al., 2017). As information on non-participants is not available, it is impossible to analyse the characteristics of those who are not drawn to these kind of interventions. However, our dropout rate was 25.1% which is relatively low compared to similar studies (Eysenbach, 2005). Moreover, as selective dropout was only present during the intervention period and for older participants, this dropout does not detract from the good generalizability of our research: the distribution of gender, educational attainment, number of comorbidities, BMI and loneliness is well in line with the average Dutch population (Dutch Department of Health Wellbeing and Sports, 2020). By performing multilevel regression analyses to the incomplete dataset, we handled missing data in the most accurate way as this method has proven to result in better estimations than using multiple imputation (Twisk et al., 2013).

Secondly, adherence to the intervention was not tested: to what extent participants actually read or used the intervention advice is thus unknown. However, studies into previous versions of the Active Plus intervention demonstrated that the printed materials were read by more than 93% of the participants (Peels et al., 2013). As participants received the intervention materials both printed and online, exposure in the present study is expected to be comparable.

Thirdly, we enhanced the scale of the loneliness questionnaire to better align with the needs of our specific target population. As these enhancements were performed in line with recommendations for designing questionnaires for older adults (Krosnick and Presser, 2010; Remillard et al., 2014), we expect that the validity of the questionnaire remains intact.

Fourthly, we categorized the degree of mobility impairment into low, medium or severe by taking the highest degree of impairment as perceived by the participant caused by any of their chronic diseases. Some studies have demonstrated that certain combinations of chronic diseases increase the total degree of mobility impairment beyond what could be expected based on the degree of impairment of the individual chronic diseases (Raina et al., 2020). Incorporating this in our questionnaire could have given a more complete insight and is thus recommendable for future studies.

## Conclusions

To our knowledge, this is the first RCT that analyses the effects of an intervention on loneliness by stimulating social activity while being physically active for the target population of older adults with chronic diseases. As societies are ageing and age is often accompanied with the onset and deterioration of both chronic diseases and loneliness, this is a target population that is growing in importance. Notwithstanding the above-mentioned limitations, our findings indicated that the Active Plus intervention was able to decrease total and social loneliness on the long term among the target population. Subgroup analyses demonstrated that especially the vulnerable age group of 80 years and older seemed to benefit more from Active Plus. For future research, it may be advisable to use a more comprehensive tool for measuring social activity and mobility impairments and to use a longer time frame in order to better detect changes in loneliness.
